# Honeyomics and Industrialisation of Madu Kelulut as a Health Supplement: Are We Ready for Scale-Up?

**DOI:** 10.21315/mjms2024.31.5.1

**Published:** 2024-10-08

**Authors:** Mohd Zulkifli Mustafa, Patricia Vit

**Affiliations:** 1 Department of Neurosciences, School of Medical Sciences, Universiti Sains Malaysia, Kelantan, Malaysia; 2 Apitherapy and Bioactivity, Food Science Department, Faculty of Pharmacy and Bioanalysis, Universidad de Los Andes, Mérida, Venezuela

**Keywords:** honeyomics, madu kelulut, commercialisation, superfood, health supplement

## Abstract

Ethnomedicinal uses of honey value its nutritional and medicinal properties to attain general health, diseases prevention or treatment for particular illness. Madu kelulut or stingless bee honey is produced by Meliponini species and the honey referred as superfood in Malaysia. The honey is a complete natural food product that provide safe source of energy via low GI trehalulose sugar, nourished with nutrients, vitamins, amino acids and good bacteria that is formed via unique natural pot-bioreactor processing. The honey is gaining attention for its high antioxidant levels, antimicrobial properties, anti-inflammatory, unique composition and taste. Studies revealed multiple foundations and evidence-based that the madu kelulut is highly beneficial for human possessing a promising niche in the health supplement industry with economic importance projected up to USD700 million in local markets. Honeyomics of madu kelulut aims at understanding the economic importance factors that contribute to the entire mark-up of madu kelulut as general guides for standards and the functional properties of madu kelulut. Several milestones (2005–2023) have been achieved which acknowledge the strategic development and ecosystem for the madu kelulut industry in Malaysia. Further efforts to safeguards the quality and authenticity of stingless bee honey via modernisation, capacity building and market expansion could encourage for scale-up and solidifying Malaysia’s position as a key player in the global stingless bee honey industry, providing economic benefits while preserving natural resources for future generations.

## Introduction

Madu kelulut or stingless bee honey is defined as a natural sweet with a certain sour substance like aliphatic organic acids produced by stingless bees (Meliponini tribe) and associated microbiota (1) from the nectar of plants or from secretions of living parts of plants, which the stingless bees collect, transform by combining with the specific substances of their own, deposit, dehydrate, transform with associated microbes, and store, in the natural honey pots to ripen and mature. Whereas the honeyomics perspective here is simply probing at the economic importance and data of honey origin, constituents and collective characterisation of biological molecules or elemental fingerprints which translate into the dynamics character of the honey. Honeyomics of madu kelulut aims at understanding the factors that contribute to the entire mark-up of madu kelulut becoming crucial foundation for standards and the functional properties of madu kelulut towards commercialisation.

Ethnomedicinal uses of honey value its nutritional and medicinal properties to attain general health, diseases prevention or treatment for particular illness. It is an ingredient of various food products as well as cosmetics, and pharmaceuticals products. The global honey market is well-established and was valued at approximately USD9 billion in 2023 and is projected to grow at a compound annual growth rate of about 7%–8% over the next several years (2). The madu kelulut market, while much smaller in comparison is a niche and rapidly growing segment within the broader honey market but projected up to USD700 million in local market driven by increasing recognition of its unique health benefits and its status as a rare and premium product, making the madu kelulut a new economically important product of Malaysia.

Despite tremendous recognition as a superfood or miracle liquid, madu kelulut is not currently a honey according to the CODEX Alimentarious honey standard that only recognises a single honeybee species (*Apis mellifera*) in the main definition of honey (3). Nevertheless, the Malaysian standard of kelulut (stingless bee) honey (specification 2683:2017) was introduced by the Department of Standards Malaysia (4) which recognised and gazetted the madu kelulut as a regulated honey. The distinctive characteristics of stingless bee honey are intricately linked to the unique honey formation process employed by stingless bees. Unlike the common stinging honeybee, stingless bees store their honey in pot-like structures made of cerumen, a mixture of stingless beeswax and plant resins. This storage method contributes to the honey’s higher moisture content, as the pots have different efficiency at moisture regulation than the wax combs used by honeybees. These bioreactors permit fermentation by microbes associated with stingless bees. The higher moisture content results in a thinner, more liquid honey that is more prone to fermentation (5) leading to a unique fruity sweet sour and tangy flavour.

This article intended to outline several factors affecting the madu kelulut formation as well as current establishment of stingless bee keeping and development in Malaysia as a foundation for the industry expansion.

### Honey as Sacred Food and Features According to Al-Quran

Ethnomedicine has been recorded as one of the important approaches in traditional health management. However, honey portrayed an outstanding symbolism. Across various religions and ancient cultures, honey has been consistently revered as a symbol of health, healing and divine favour. Its frequent mention in sacred texts highlights not only its practical uses but also its symbolic importance, representing purity, prosperity and the sweetness of life. This deep cultural and religious significance has contributed to honey’s esteemed status throughout history, making it a timeless and universal symbol of nourishment and well-being.

The Quran explicitly mentions honey in Surah An-Nahl (The Bee), 16:68−69: “And your Lord inspired the bee, saying: Take your habitations in the mountains and the trees and in what they erect. Then, eat of all fruits, and follow the ways of your Lord made easy for you. There comes forth from their bellies, a drink of varying colour wherein is healing for mankind. Verily, this is indeed a sign for people who think” (6). It is clearly known to Muslims that the Quran revelation is embodies to all mankind, eras and places. These verses have set a standard of honey definition that should become a major guide to understand the holistic of honey formation and application.

Both Apini (honeybee) and Meliponini (kelulut) are Hymenopteran tribes in the family Apidae, subfamily Apinae. However, the oldest fossil of a bee is a Meliponini up to 96 million years old that is much older than honeybee. Both bees can be found nesting at the mountains, trees and manmade buildings. The fact that honey is solely produced by bees was validated via the special honey stomach discovered within the bee bellies and also producing honey in various colours that is proven with remedy properties. In the Quran verse, there is no indication that the bee must have a sting or originate from particular continents or criteria of honey-holding comb allows for both bee species enclosed within the verse. However, the verse is specifically highlight bees should eats fruits in which commonly represents the Meliponini foraging behaviour. Further interesting fact from the verse, the output of bee bellies is denoted as ‘a drink’ instead of referring to as honey in which open for interpretation that the honey should be taken in a high volume drink or it is referring to higher moisture content of the bee output. Higher moisture content is the major difference between the stingless bee honey and the honey from honeybee. All these facts highlighting that the madu kelulut is a type of honey which align with the guidelines by the Quran and perhaps, the primary remedy honey.

### Honeyomics of Madu Kelulut

Internationally, madu kelulut often referred to as ‘meliponini honey’ or ‘pot-honey’, is characterised by its unique cerumen pot storage, composition or properties that distinguish it from honey produced by *Apis mellifera*, the common honeybee.

One of the prominent features of madu kelulut is its higher moisture content, which typically ranges between 25%–35%, compared to the 17%–20% moisture content of honeybee honey. This elevated moisture level contributes to its thinner consistency and slightly fermented, which is often described as more acidic. In Malaysia, high humidity and relatively big sizes of honey pots further increase the moisture content in madu kelulut and promote fermentation which increases the free acidity and produces a much sour taste.

With 605 species of stingless bees identified in tropical or subtropical regions (7), the foraging behaviour of stingless bees in a biodiverse environment, adds more variation in honey composition. These factors can be broadly categorised into resource factors, biological activities in pot involving bee’s gut microbiome and also stingless bee keeping practices or handling factors that contribute to the final product’s characteristics.

#### Resource Factors

Stingless bees typically forage on a wide variety of plants determining the quality of honey. The botanical origin significantly affects honey’s smell, flavour, colour and overall composition. Honey derived from a single type of flower, known as monofloral honey, tends to have a more consistent quality. In contrast, polyfloral honey, which comes from the nectar of various flowers, can exhibit greater variability. The fact that the stingless bee is of variable size and foraging on fruits, plant sap and nectars of small flowers, is believed to increase the richness of components in the madu kelulut including the phytochemicals (8). Phytochemicals are secondary metabolites, naturally occurring compounds found in plants and pollen, including flavonoids, phenolic acids, polyphenols and other bioactive substances that are being transferred from plants to the honey pots. This richness is further influenced by geographical factors such as climate, weather conditions and soil composition. The climate affects the humidity and types of flowering plants, while soil nutrients can impact plant growth and nectar production, indirectly influencing honey quality.

#### Activities in Cerumen Pots

Stingless bees store their honey in a unique cerumen pot developed from plant resins and complex admixtures of stingless beeswax, and plant exudates with its saliva. Plant resin contains a variety of bioactive compounds, including flavonoids, phenolic acids and essential oils. The specific composition of these resins depends on the plant species from which they are collected. Small amounts of bee pollen and micro-wooden chips are often mixed during pots construction. Resources like nectar is later stored in the pot to form the honey. As the honey is stored in these pots, it comes into contact with the propolis, allowing the bioactive compounds from the resin to infuse into the honey. The presence of lactic acid bacteria (LAB) and yeast lining on the inner wall of the pots have created the natural bioreactor-like pots which add another layer of complexity to their honey.

The sugar compositions of stingless bee honey are diverse according to the primary source of nectars and further sugar digestion by LAB present in the bees’ stomachs. These bacteria contribute to the bees’ health and the initial stages of honey preservation. The metabolic products from LAB, such as aliphatic organic acids, can influence the flavour and acidity of honey (9). Apart from the microbial flora, multiple predominant enzymes such as diastase, invertase, glucose oxidase, catalase and phosphates that are highly present in the bees’ stomachs also play a significant role in transforming nectar into honey. For instance, the enzymatic invertase contributes by hydrolysing the nectar sucrose into two major monosaccharides, namely fructose and glucose. The glucose is further converted into gluconolactone and later forms hydrogen peroxidase via glucose oxidase enzyme that is derived from the salivary gland of stingless bees. This hydrogen peroxidase acts as a potent antimicrobial agent that enhance honey’s preservative qualities and effectively inhibits the growth of microbes within the honey (10). Yeasts may be present in small quantities and can survive in the acidic, low-water environment of honey. They can ferment sugars, but their activity is generally minimal due to honey’s low moisture content in honeybee honey. In light of the stingless bee honey possessing higher water content, the yeast dominates by performing fermentation that further yields alcohol, carbon dioxide and bioactive substances (e.g. aliphatic organic acids and short-chain fatty acids). They eventually contribute to increase acidity in the honey and enhance various beneficial properties of the stingless bee honey, including antimicrobial, antioxidant, anti-inflammatory and immune-modulating effects that further support the overall health and immunity system.

Interestingly, stingless bee honey is highly rich in the novel natural occurrence of trehalulose sugar that associate with low glycaemic index (GI). The trehalulose holds much slower rate of release of monosaccharides into bloodstream than sucrose and also possesses highly active antioxidant (11).

While nectar is the primary source of sugars in honey, pollen contributes additional bioactive compounds, including proteins, vitamins, minerals and a variety of phytochemicals (12). Once collected by bees, pollen undergoes a natural fermentation process facilitated by enzymes and microorganisms present in the hive. During fermentation, the complex carbohydrates and proteins in pollen are broken down into simpler forms and phytochemicals are released or transformed into more bioactive forms which enhance the bioavailability of these compounds (13) and potentially contributes to the unique medicinal properties of stingless bee honey.

Dynamic activities of these factors highly define the nutritional composition of the honey that contributes to the honey’s health benefits.

#### Handling Factors

The honey quality is not only determined by the biological aspect of pots and the activities of bees but beekeeping practices also introduce another layer of complexity to pot-honey. The methods used in processing and storing honey, including extraction, filtration and heat treatment, can significantly impact its natural volatile compound, enzymes, nutrients and flavour. For example, excessive heat treatment may reduce enzyme activity and alter the nutritional profile, leading to a decline in quality. Stingless bee keeping practices, including hive management and the use of chemicals to control pests and diseases, can also influence honey quality. Meeting regulatory and quality standards amidst this variability is challenging, as it requires consistent monitoring and control across different batches of pot-honey.

Overall, understanding to all variables is essential for ensuring pot-honey quality and meeting both regulatory standards, consumer expectations and most importantly its value as a remedy substance. More research is needed to establish standardised guidelines for the therapeutic use or application of madu kelulut especially for emerging areas like edible vaccines and gut-microbiome for health. Current studies often vary in their methodologies, making it difficult to draw definitive conclusions about the optimal dose. Nevertheless, clinical observation in pre-diabetic patients (*n* = 30) indicated that 30 g of madu kelulut consumption for 30 days did not increase the blood sugar levels (14).

### Scale-Up of Madu Kelulut in the Health Supplement Industry

Studies revealed multiple foundations and evidence-based that the madu kelulut is highly beneficial for human (15) possessing a promising niche with significant growth opportunities for the health supplement industry.

**Figure 1:**
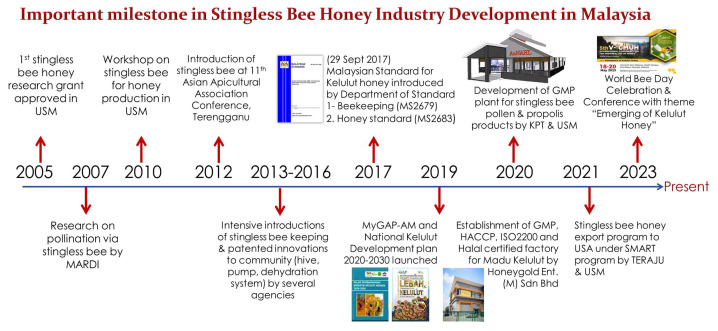
Summary of historical background and activities in the past 18 years of stingless bee honey industry development in Malaysia

**Figure 2:**
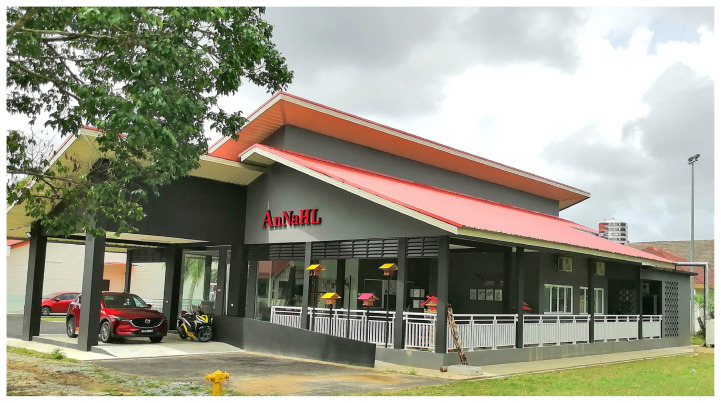
The Rumah Kelulut AnNaHL established at Universiti Sains Malaysia in 2023

This is further supported by ecosystem development of the madu kelulut industry in Malaysia, as summarised in Figure 1 below. Several milestones (2005–2023) have been achieved as a foundation and acknowledgement that the madu kelulut industry in Malaysia arouse with strategic planning and collaboration between government agencies, universities, enterprises, along with local communities of stingless bee keepers. Figure 2 showed a new landmark named Rumah Kelulut AnNaHL (Advance National Honey Landmark) in Universiti Sains Malaysia established under the Translational Research Grant Scheme, Ministry of Higher Education (MoHE) Malaysia. The existing Rumah Kelulut AnNaHL is an inspiration to transform the local traditional pot-honey industry to another level and currently functioning as one stop centre for stingless bee honey research that equipped with good manufacturing practices (GMP) facilities.

Furthermore, the current status indicates that the madu kelulut is the emerging industry and shows a potential to be a new agricultural commodity that can drive Malaysia economy according to multi-dimensional parameters summarised in Table 1.

## Conclusion

Madu kelulut is a complete natural food product that provide safe source of energy via low GI sugar, nourished with nutrients, vitamins, amino acids and LAB that is formed via unique pot processing of several botanical resources. As the output, madu kelulut is gaining attention for its higher antioxidant levels, antimicrobial properties, anti-inflammatory, unique composition and taste, making it appealing to health-conscious consumers and those interested in natural remedies. Honeyomics of madu kelulut presented the economical important insight and mass biological inputs that highlight the fermentation occurrences and increased the nutritional values in madu kelulut, in which the sweet-sour characteristic as a result of the fermentation should be recognised as an advantage and natural taste. As a further effort to capitalise this industry, it is recommended that all stakeholders need to focus on market education, product innovation and positioning stingless bee honey as a premium health supplement. Efforts to safeguards the quality and authenticity of stingless bee honey via modernisation, capacity building and market expansion could encourage for scale-up and solidifying Malaysia’s position as a key player in the global stingless bee honey industry, providing economic benefits while preserving natural resources for future generations.

## Figures and Tables

**Table 1:** Multi-dimensional parameters as readiness foundation for madu kelulut to become a new agricultural commodity for Malaysia

No.	Parameters	Status
1.	Agronomic feasibility	The stingless bee farming in Malaysia is steadily growing since 2012, involving farmers from diverse backgrounds, achieving honey yields of 3 kg–5 kg per colony established within rubber and coconut plantations, acacia forests and local orchards. Two main productive species are the *Heterotrigona itama* and *Geniotrigona thorasica*. The production cycle, is typically running from March to September every year, and at present becomes a viable supplementary income estimated at RM2,135.39 monthly (16) to stingless bee keepers.
2.	Climatic and environmental suitability	The kelulut deep-rooted history in Malaysia with more than 30 species were found thriving in the tropical forests, well-adapted to the high humidity and warm climate supporting colony growth, making them an integral part of Malaysia’s forest and biodiversity.
3.	Economic viability	Stingless bee products have shown its competitiveness to compete within global honey market and locally was valued at approximately RM3 billion. The honey demand is significantly increase driven by consumer interest in natural, health-focused products and distinctive flavour. Of particular interest is the presence of trehalulose, a rare sugar with a low glycaemic index (GI), found in stingless bee honey that sparked new interest in the honey for alternative healthy sugar. With the growing awareness of its benefits and the potential for expanding production, stingless bee honey is sells between RM270—RM500 per kilogram and becoming profitable crop that holds promise as a valuable export commodity.
4.	Socioeconomic impact	The stingless bee honey industry has significant socioeconomic impacts, particularly in job creation and community development. The growth creates opportunities in other sectors, including farming, honey processing, packaging, marketing, and related industries like equipment manufacturing and meli-tourism. These provide direct employment and improving livelihoods in which the readiness of local communities to adopt the industry is at peak. The industry also encourages collaboration between universities, government agencies, and private enterprises particularly in R&D and regulating the industry, providing training, certifications, support to farmers and positively foster not only economic development but also environmental sustainability.
5.	Technology and innovation	Local stingless bee honey industry is experiencing significant advancements in technology which are enhancing efficiency and sustainability. One notable development is the introduction of patented hive designs, which optimise bee management and honey collection. Honey dehydration systems are being implemented to preserve honey quality and extend its shelf life while in vitro colony production techniques are also emerging, allowing for the controlled breeding of stingless bees, which supports the scalability of bee populations and honey production. Mechanisation is further advanced by the use of harvesting pumps and quality measurement kit, which streamline the honey extraction process. These technologies are complemented by the growing need for a robust tracing system, with Internet of Things (IoT) and blockchain technology being increasingly considered to ensure transparency and traceability in the supply chain. These innovations collectively contribute to the industry’s growth, making stingless bee honey farming more sustainable, efficient, and profitable.
6.	Market linkages and value chain development	Market linkages and value chain development are critical to ensure the stingless bee product reaches consumers efficiently and profitably. Improved infrastructure and logistics that facilitate the smooth movement of honey includes efficient transportation networks, well-organised distribution channels, and strategic partnerships with retailers and exporters were designed particularly from Sarawak to Malaysian Peninsular via TERAJU-USM Project. Beyond raw honey, the development of diverse products such as honey-based cosmetics, health supplements and gourmet food items has opened new market segments. Processing innovations, such as honey dehydration and flavour enhancement, further increase the appeal and shelf life of stingless bee honey. Packaging that adds value, with premium, eco-friendly designs already attracting higher-end markets.
7.	Infrastructure and logistics	The growth and efficiency of the stingless bee honey industry currently supported by the development of several honey collection centres by government agencies and also establishment of specialised facilities by local enterprise, such as ‘kelulut banks,’ that has been crucial for the storage and bulk supply. These facilities are equipped with innovations to maintain honey quality, prevent contamination and extend shelf life under the GMP, Hazard Analysis and Critical Control Points (HACCP) and Halal certified plant. Current infrastructures streamline the logistics of honey collection and distribution to meet growing demand.
8.	Policy and regulatory environment	The regulatory ecosystem for the stingless bee honey industry in Malaysia is well-structured, providing a solid foundation for its growth. A key milestone is the recognition of ‘madu kelulut’ through the Malaysian Specification 2683:2017, established by Jabatan Standard Malaysia with the guidelines for honey quality, safety and authenticity. The MyGAP-AM (Malaysian Good Agricultural Practices – Apis spp. and Meliponini) certification by the Department of Agriculture as well as implantation of the GMP, HACCP, ISO22000, Halal and MeSTI certifications reinforces the quality assurance at farming level. Various government bodies, including SIRIM, MARDI, FELDA, RISDA, TERAJU, FRIM and local universities, have introduced numerous incentives programmes to support stingless bee keepers which providing financial aid, technical training and research support that facilitating the policy framework and ensure the honey meets national and international agricultural standards and regulations.
9.	Sustainability	The long-term viability of the stingless bee honey industry in Malaysia is assured via the innovations and sustainable practices. The expansion of the industry once faced challenges, particularly with the acquisition of colonies from the wild and created a threat as feral colony hunting led to a decline in wild bee populations. Current solutions like in vitro colony production have emerged as a key method for sustainable cultivation, alongside with queen breeding programmes and the use of swarm traps have empowered stingless bee keepers to increase colony production at the industry’s scalability. The stingless bees also play a crucial role in pollination, enhancing biodiversity and supporting in diverse agricultural settings, such as orchards and forests, contributes to the vitality of both cultivated and wild plants.
10.	Risk assessment	One of the primary concerns is the threat of adulterated honey inflowing the market. The presence of counterfeit honey can significantly damage consumer trust and perception, even if prices and demand for genuine stingless bee honey remain stable. This risk underscores the importance of rigorous quality control measures to protect the integrity of the industry. Besides, climate change also poses a significant risk where changes in temperature, rainfall patterns and the frequency of extreme weather events can affect the performance of stingless bees, potentially disrupting their foraging behaviour, reducing honey yields and its quality. Climate-related phenomena like ElNiño previously led to the outbreaks of Black Soldier Fly attack and colony decease in high density farm setup which require adaptive strategies to ensure the resilience of the stingless bee honey industry in Malaysia.
